# *%svy_freqs:* A Generic SAS Macro for Creating Publication-Quality Three-Way Cross-Tabulations

**DOI:** 10.5334/jors.318

**Published:** 2021

**Authors:** JACQUES MUTHUSI, PETER W. YOUNG, SAMUEL MWALILI

**Affiliations:** Division of Global HIV and Tuberculosis, U.S. Centers for Disease Control and Prevention, Nairobi, Kenya; Division of Global HIV and Tuberculosis, U.S. Centers for Disease Control and Prevention, Maputo, Mozambique; Strathmore University, Nairobi, Kenya

**Keywords:** SAS macro, disease prevalence, three-way cross-tabulations, reproducible research, replication-based variance estimation

## Abstract

Cross-tabulations are a simple but important tool for understanding the distribution of socio-demographic characteristics among participants in epidemiological studies. We developed a generic SAS macro, ***%svy_freqs,*** to create publication-quality tables from cross-tabulations between a factor and a by-group variable given a third variable using survey or non-survey data. The macro also performs two-way cross-tabulations and provides extra features not available in existing procedures such as ability to incorporate parameters for survey design and replication-based variance estimation methods, performing validation checks for input parameters, transparently formatting variable values from character into numeric and allowing for generalizability. We demonstrate the macro using the 2013–2014 National Health and Nutrition Examination Survey (NHANES), a complex survey designed to assess the health and nutritional status of adults and children in the United States.

## (1) OVERVIEW

### INTRODUCTION

Cross-tabulations are a basic but important tool for understanding the distribution of socio-demographic characteristics among study or survey participants in the fields of epidemiology and disease surveillance. They are useful especially when comparisons need to be performed separately for different levels of a by-group variable such as a key demographic characteristic, e.g., sex, or an outcome status such as positive or negative test result for a disease. Cross-tabulations can be even more informative if one is interested in the distribution of disease prevalence among selected factor variables (table rows) and a by-group variable (table columns). This is useful in cases where the association between disease prevalence and risk factors or exposures needs to be stratified, for instance, by sex or geographic region.

Almost all available statistical analysis software can easily perform cross-tabulations, however, output from these must be processed further to make them readily available for review and use in a publication. In Stata, one can use the table and tabulate [1] commands or Stata user’s community-contributed programs like tabout [2] or tabmult [3]. In SAS, there exist a limited number of commands or macros for creating publication-quality tables [4–9] but they suffer from limitations of flexibility, usability and generalizability. In particular, the SAS macros available do not provide the analyst with options for specifying replication-based variance estimation methods including Jackknife (JK) or Balanced Repeated Replication (BRR) which are often used in order to obtain correct variances for survey estimates in presence of survey non-response, hence providing valid variance estimates [10–12].

We have developed a SAS macro which overcomes the described shortcomings while promoting reproducible research principles [8] such as transparency, reproducibility and reusability, which are attracting increasing attention in epidemiological research [13–17]. It further provides for replication-based variance estimation methods as well as enforced validation checks for input parameters.

The work presented here builds on the development of another SAS macro, ***%svy_logistic_regression,*** for producing publication-quality tables from unadjusted and adjusted logistic regression analyses [18].

### IMPLEMENTATION AND ARCHITECTURE

#### The *%svy_freqs* SAS macro

This macro, written in SAS software version 9.3 [19], uses the SURVEYFREQ and SURVEYMEANS procedures to perform the cross-tabulation and output frequencies, totals and percentages. The macro uses the SAS output delivery system (ODS) to create a publication-quality table, similar to a typical Tables 1 or 2 of a manuscript in the epidemiological research field.

The macro is composed of seven sub-macros, which are called within the main macro. The _outcome and _outvalue, which are the parameters for which prevalence is to be computed, must be specified. Analysis type, _cat_type, must be specified as equal to PREV. If not specified, the macro automatically generates a new variable, _freq whose value equals 1 for all study subjects in the analysis dataset, and proceeds with the analysis as though it were for two-way cross-tabulations with row percentages. The _outcome and _outvalue parameters may be omitted for two-way cross-tabulations. The macro enforces in-built SAS validation checks on input parameters and tests for logical errors. It halts the macro from execution and prints out the error on the log window for the user to address. The user should specify input parameters that are described in Table 1 unless the description is prefixed by (optional). To achieve full potential of the SAS macro, the user must ensure that the analysis dataset is clean, analysis variables are well labelled, and values of variables have been converted into appropriate SAS formats before they can be input to the macro call.

Two-way cross-tabulations are also possible. For instance, if users are interested in showing distribution of study participants by a given by-group variable, then column percentages which are most appropriate are obtained using the COL option. If the by-group variable is an outcome of interest such as positive or negative diagnostic test results, then the row percentages are most appropriate and can obtained using the ROW option. The by-group variable can have more than two categories and can be encoded as either a numeric or character variable. For the distribution of continuous variables, one can specify the type of statistic to compute (mean or median).

Where the data to be analyzed come from a complex survey, our macro allows users to specify study design variables containing strata, cluster, and design weights as well as the variance estimation method and replicate weight variables, if necessary. Data from non-survey settings are analyzed by leaving the survey-design parameters unspecified. The macro also provides for domain analysis for sub-populations, and there are options for specifying how missing values should be represented [11, 20–22].

If the analysis includes non-coded character variables, the macro automatically encodes them into numeric variables prior to analysis. The macro further provides natural display of results from epidemiological surveys by processing the final output into a refined publication-quality table, which is output into word processing and spreadsheet programs for immediate use in publications or for additional formatting if needed.

The macro has several limitations. First, it has been developed on Microsoft Windows and code adjustments may be needed to adapt it for other operating systems. Second, it cannot handle arbitrary nesting of by-group variables, such as those supported by PROC TABULATE. Additionally, it does not provide interpretation of results, so users should consult a qualified statistician for any inference. Nonetheless, we feel this macro provides a good tradeoff between simplicity and ease of use, flexibility, and generalizability, and should shorten the analysis period for complex surveys, while supporting generation of high-quality outputs.

#### Quality Control

##### Example of macro call to analyze the NHANES dataset

We demonstrate the application of the macro in the analysis of a dataset from the 2013–2014 National Health and Nutrition Examination Survey (NHANES). NHANES is a complex survey designed to assess the health and nutritional status of adults and children in the United States (U.S.). A detailed description of the survey design and contents is available elsewhere [23]. The NHANES dataset [24] is publicly available online for free from the U.S. Centers for Disease Control and Prevention (CDC) at: https://www.cdc.gov/nchs/nhanes/Index.htm. Data used for this demonstration is also available at the GitHub repository (https://github.com/kmuthusi/three-way-crosstabulation-macro)

We used the macro to generate three different tables with the main one (Table 4 with prevalence percentages) showing the distribution of hepatitis A prevalence across selected socio-demographic characteristics and by sex. The next tables show the distribution of participants’ socio-demographic characteristics by sex (Table 2 with column percentages) and by hepatitis A antibody test result (Table 3 with row percentages). The aim of the analysis was to show the distribution of hepatitis A among participants aged 20+ years who had served active duty in the U.S. Armed Forces. We also show participant’s socio-demographic characteristics by sex and by hepatitis A antibody test result. Appropriate survey weights (sample weights for participants with a medical examination) were applied. The working denominator was N = 542. However, 180 observations were dropped during analysis because they had non-positive weights. In addition, the analysis domain sample size was calculated and added at the end of each table title.

The macros were run sequentially after specifying required parameters as shown in code Examples 1–3 The results presented here are purely for illustrative purposes only and do not follow from any specific survey objective. Readers should consult the NHANES analytic guidelines on variable definitions, analytical and statistical recommendations that are available online at https://wwwn.cdc.gov/nchs/nhanes/analyticguidelines.aspx.

The SAS output from the macro consists of several tables specifically for holding parameter estimates, corresponding 95% CI for percentages and means or IQR for median. Table 2 displays distribution of patient characteristics (row variables) by sex (column variable) which was output after running the code in Example 1. Columns include categories and factor labels in the first column, followed by unweighted sample size for each level of the factor, weighted column percentages/or median and corresponding 95% CI or IQR. To compare the distribution of selected factors by sex, we use the 95% CI or IQR. For instance, among participants aged 40–59, there were more females than males 61.8% (95% CI: 38.2–85.3%) versus 25.2% (95% CI: 19.9–30.5%). Median age at screening was comparable at 64.2 years (IQR: 51.3–73.0) for males compared to 50.3 years (IQR: 41.1–53.8).

Table 3 shows the distribution of patient characteristics by hepatitis A test results which was obtained after running the code in Example 2. The output presents row percentages and includes columns for missing values. The results show the distribution of hepatitis A status across the given factor variables. It can be seen that there are no significant differences in the distribution of hepatitis A status with 37.1% (95% CI: 33.6–40.6%) males and 38.9% (95% CI: 17.3–60.4%) for females reporting positive status, though there are differences between specific age groups. It is important to note that if missing values are suppressed the estimates will also change since the denominator will have changed.

Table 4 shows distribution of hepatitis A prevalence by sex obtained after running the code in Example 3. The columns in Table 4 are also organized in a similar way as described for the previous tables. The output shows there were no significant difference across each factor by sex as the confidence intervals are overlapping due to the small sample size of females.

The macro has been extensively tested by the developer by comparing output to direct tabulation in the underlying SAS software. If desired, the end user can also request the NHANES dataset from CDC in order to reproduce the analyses in this paper to confirm the correct operation of the macro on their system by using the corresponding analysis and output files provided in the GitHub repository (https://github.com/kmuthusi/three-way-crosstabulation-macro).

## Figures and Tables

**Example 1:**
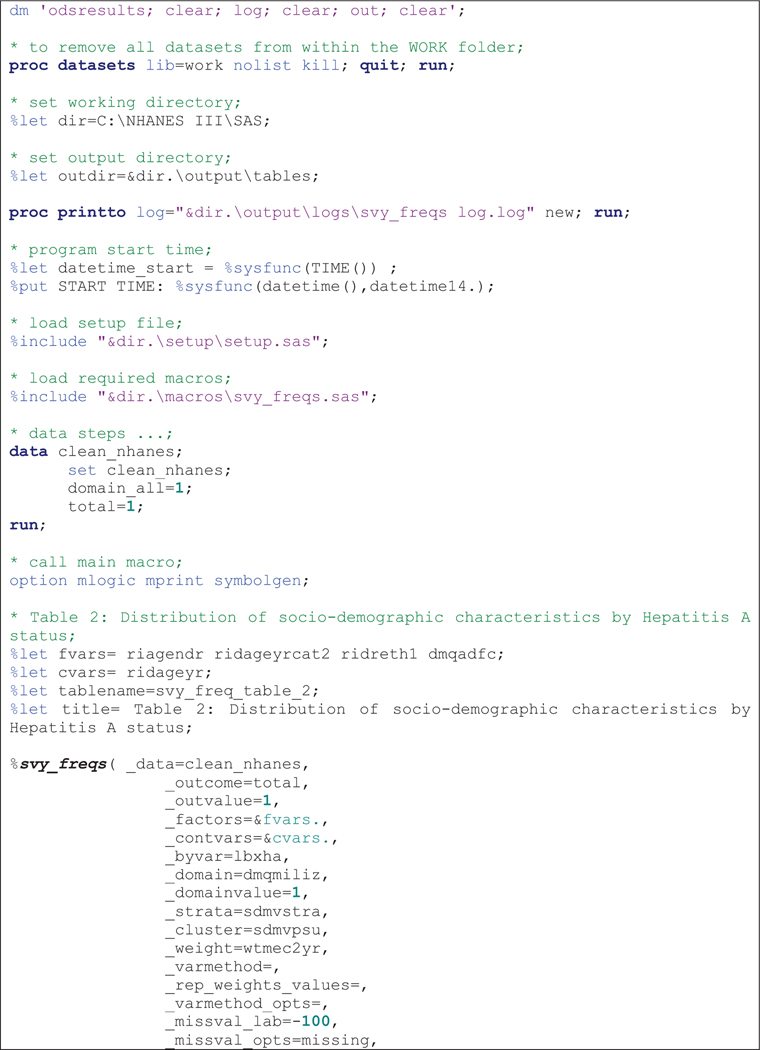

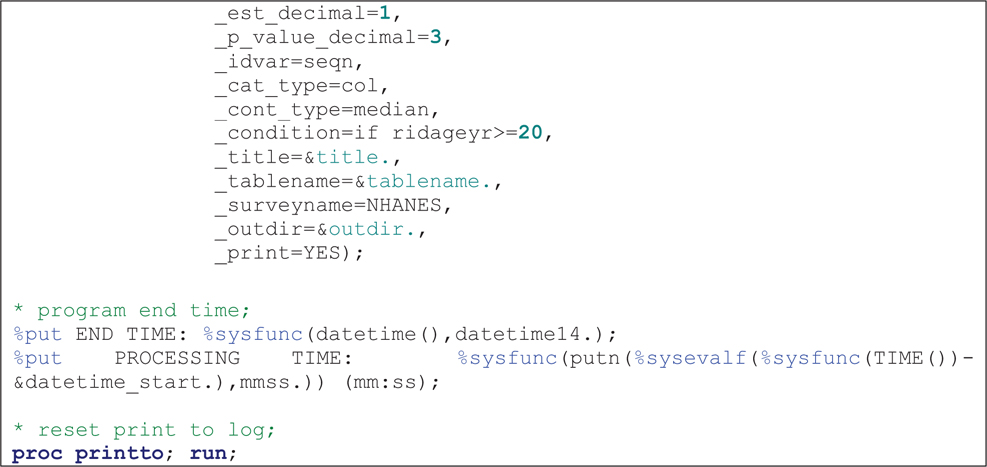
Sample *%svy_freqs* macro call to output column percentages.

**Example 2:**
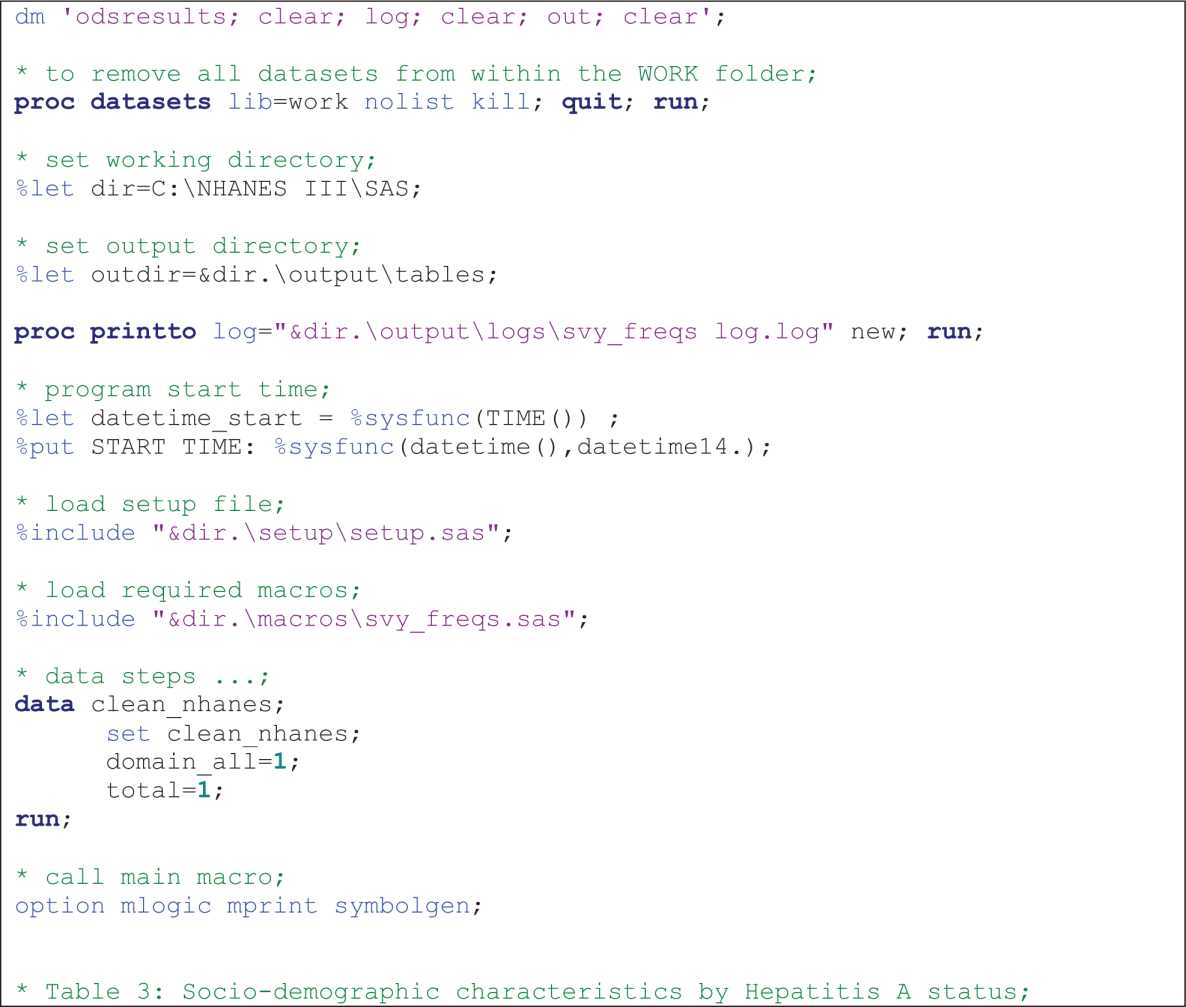

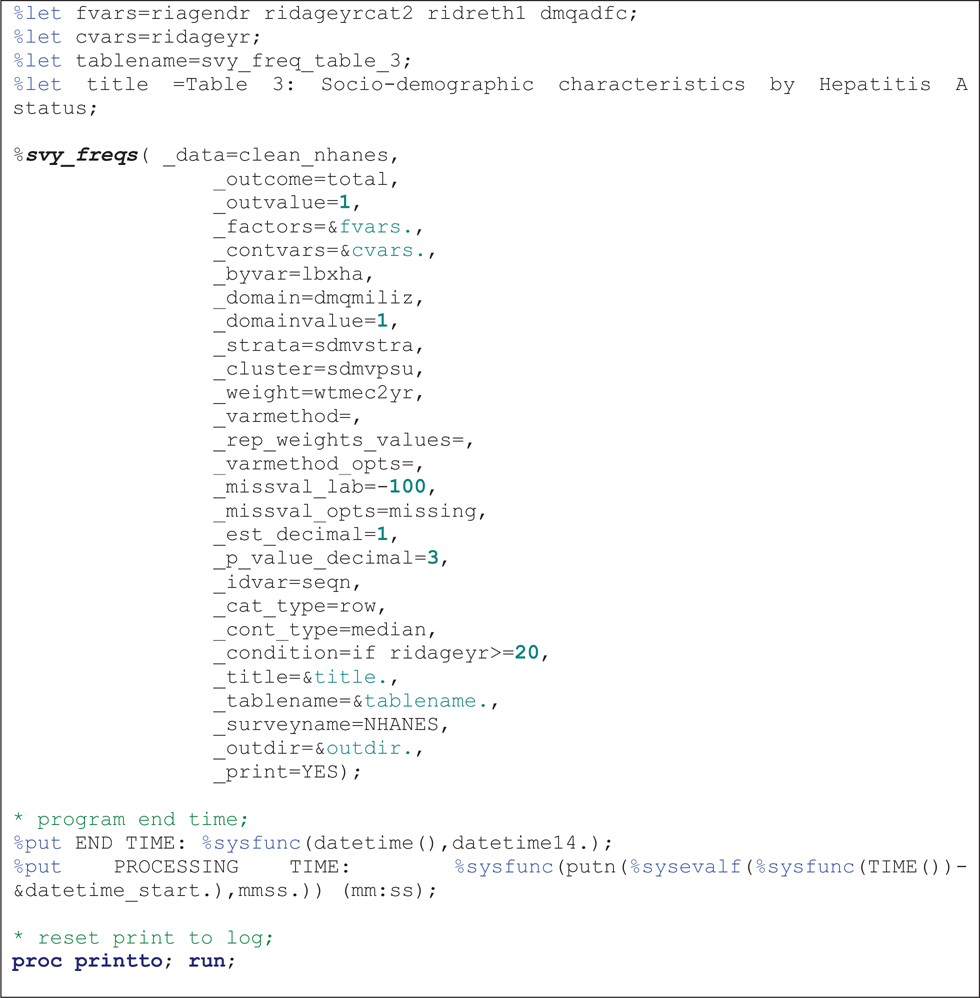
Sample *%svy_freqs* macro call to output row percentages

**Example 3:**
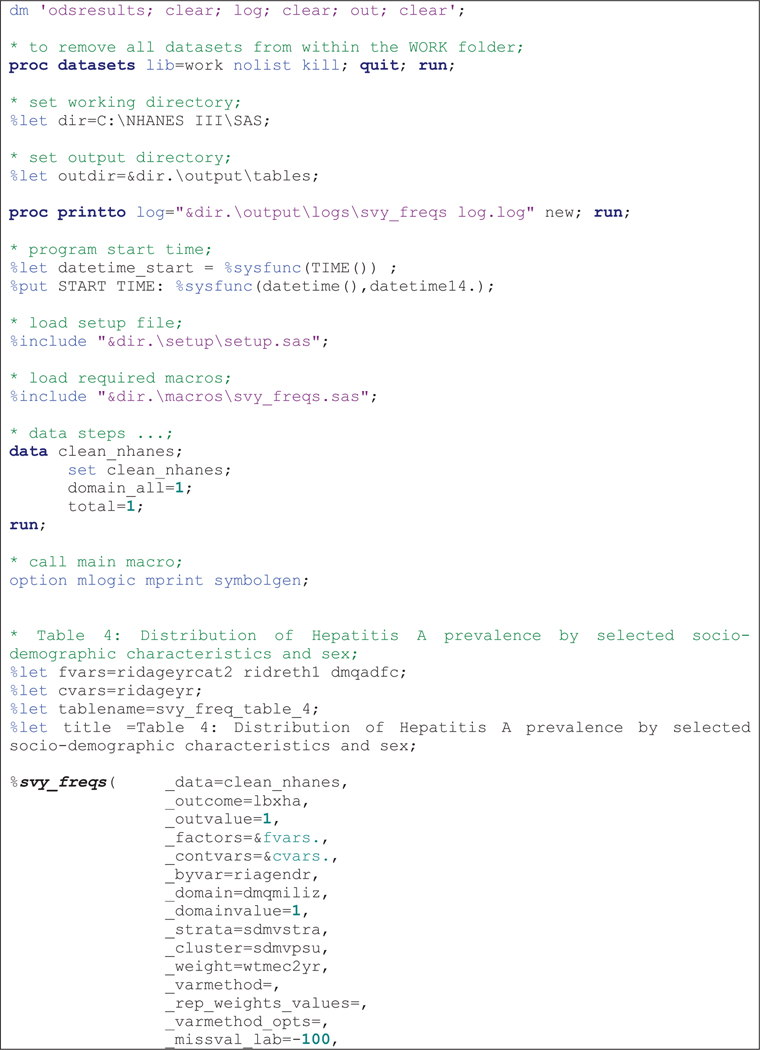

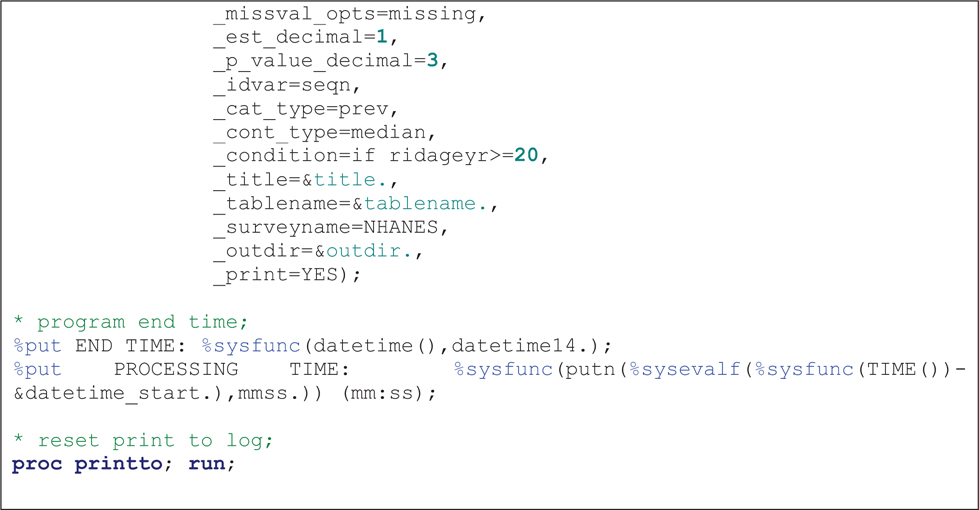
Sample *%svy_freqs* macro call to output prevalence percentages.

**Table 1:** Input parameters for *%svy_freqs* macro.

PARAMETER	DESCRIPTION
_data	name of input dataset
_factors	list of categorical variables separated by space
_cat_type	type of analysis for categorical variables i.e., COL for column percentages, ROW for row percentages, PREV for prevalence percentages
_contvars	list of continuous variables separated by space
_cont_type	type of analysis for continuous variables i.e., MEAN or MEDIAN
_byvar	name of categorical by-group variable which can have any number of categories/levels
_outcome	(optional) name of third variable for which cross-tabulations are needed e.g., lbxha, for Hepatitis A, but must be specified if prevalence analysis is being performed
_outvalue	(optional) value label of third variable to compute prevalence cross-tabulation but must be specified if _outcome is specified e.g., Positive, in the case of prevalence of Hepatitis A.
_strata	(optional) survey stratification variable
_cluster	(optional) survey clustering variable
_weight	(optional) survey weighting variable
_domain	(optional) domain variable for sub-population analysis
_domainvalue	(optional) value of domain/sub-population of interest (should be numeric). Required if _domain is specified
_varmethod	(optional) value for variance estimation method namely Taylor (the default) or replication-based variance estimation methods including JK or BRR
_varmethod_opts	(optional) options for variance estimation method, e.g., jkcoef = 1 df = 25 for JK
_rep_weights_values	(optional) values for REPWEIGHTS statement, but may be specified with replication-based variance estimation method is JK or BRR
_missval_lab	(optional) value label for missing values. If missing data have a format, it should be provided, otherwise macro assumes the default format “.”
_missval_opts	(optional) options for handling missing data within proc survey statement, e.g., “MISSING” or “NOMCAR”. If no option is specified all missing observations are excluded from the analysis
_idvar	name of unique identifying variable
_condition	(optional) any conditional statements to create and or fine-tune the final analysis dataset specified using one IF statement
_outdir	path for directory/folder where output is saved
_tablename	short name of output table
_tabletitle	title of output table
_surveyname	abbreviation for survey/study to be included in the output
_print	variable for displaying/suppressing the output table on the output window which takes the values (NO = suppress output, YES = show output)

**Table 2:** Participants’ socio-demographic characteristics by sex (Col %), N = 522^π^

CHARACTERISTIC^£^	MALE			FEMALE			TOTAL^€^		
	UNWEIGHTED N^@^	WEIGHTED % (OR MEDIAN)^¥^	95% CL (OR IQR)^$^	UNWEIGHTED N	WEIGHTED % (OR MEDIAN)	95% CL (OR IQR)	UNWEIGHTED N	WEIGHTED % (OR MEDIAN)	95% CL (OR IQR)
Age in years at screening									
20–39	46	12.7	(7.9–17.5)	6	19.2	(2.8–35.6)	52	13.3	(8.6–18.0)
40–59	114	25.2	(19.9–30.5)	24	61.8	(38.2–85.3)	138	28.3	(21.4–35.2)
>= 6060	326	62.1	(56.0–68.1)	6	19.1	(2.2–35.9)	332	58.4	(51.5–65.4)
Total	486	100	(__–__)	36	100	(__–__)	522	100	(__–__)
Race/Hispanic origin									
Mexican American	23	2.6	(0.7–4.5)	0	.	(.–.)	23	2.4	(0.7–4.1)
Other Hispanic	25	3.2	(1.6–4.8)	2	2.9	(0.0–7.5)	27	3.2	(1.5–4.8)
Non-Hispanic White	296	80.1	(74.0–86.2)	16	68.3	(52.4–84.3)	312	79.1	(73.1–85.2)
Non-Hispanic Black	115	10.2	(6.4–14.0)	17	27.7	(14.3–41.1)	132	11.6	(7.8–15.5)
Other Race	27	3.9	(1.5–6.2)	1	1.1	(0.0–3.6)	28	3.6	(1.5–5.8)
Total	486	100	(__–__)	36	100	(__–__)	522	100	(__–__)
Served in a foreign country									
Yes	261	52.4	(46.2–58.6)	14	36.8	(18.7–54.9)	275	51.1	(45.7–56.5)
No	224	47.6	(41.4–53.8)	22	63.2	(45.1–81.3)	246	48.9	(43.5 –54.3)
Missing	1	0.6	(0.0–2.0)	0	.	(.–.)	1	0.6	(0.0–1.8)
Total	486	100	(__–__)	36	100	(__–__)	522	100	(.–.)
Hepatitis A antibody									
Positive	205	37.7	(34.0–41.4)	15	39.7	(18.0–61.3)	220	37.9	(34.8–40.9)
Negative	268	62.3	(58.6–66.0)	20	60.3	(38.7–82.0)	288	62.1	(59.1–65.2)
Missing	13	1.7	(0.7–2.7)	1	2.1	(0.0–6.0)	14	1.7	(0.8–2.7)
Total	486	100	(__–__)	36	100	(__–__)	522	100	(__–__)
Median age in years at screening	486	64.2	(51.3–73.0)	36	50.3	(41.1–53.8)	522	63.5	(49.5–72.2)

π = Analysis domain sample size.

€ = By-group variable.

£ = column for listing factor variables labels and corresponding categories.

@ = column for listing raw frequencies.

¥ = column for listing weighted column/row/prevalence % (for categorical factors) or median/mean (for continuous factors).

$ = column for weighted 95% confidence interval (for column/row/prevalence/mean % estimates) or interquartile range, IQR (for median estimates).

**Table 3:** Socio-demographic characteristics by Hepatitis A status (Row %), N = 522.

CHARACTERISTIC	POSITIVE			NEGATIVE			MISSING		
	UNWEIGHTED N/N	WEIGHTED % (OR MEDIAN)	95% CL (OR IQR)	UNWEIGHTED N/N	WEIGHTED % (OR MEDIAN)	95% CL (OR IQR)	UNWEIGHTED N/N	WEIGHTED % (OR MEDIAN)	95% CL (OR IQR)
Gender									
Male	205/486	37.1	(33.6–40.6)	268/486	61.2	(57.3–65.2)	13/486	1.7	(0.7–2.7)
Female	15/36	38.9	(17.3–60.4)	20/36	59.0	(38.2–79.9)	1/36	2.1	(0.0–6.0)
Total	220/522	37.2	(34.4–40.1)	288/522	61.0	(57.7–64.4)	14/522	1.7	(0.8–2.7)
Age in years at screening									
20–39	39/52	82.4	(72.6–92.2)	12/52	16.9	(7.1–26.7)	1/52	0.7	(0.0–2.3)
40–59	50/138	30.8	(23.1–38.5)	85/138	67.9	(59.7–76.1)	3/138	1.3	(0.0–2.6)
>= 6060	131/332	30.1	(25.3–34.9)	191/332	67.7	(62.4–73.1)	10/332	2.2	(0.4–3.9)
Total	220/522	37.2	(34.4–40.1)	288/522	61.0	(57.7–64.4)	14/522	1.7	(0.8–2.7)
Race/Hispanic origin									
Mexican American	14/23	67.1	(41.3–92.8)	9/23	32.9	(7.2–58.7)	0/23	.	(.–.)
Other Hispanic	17/27	62.1	(30.6–93.6)	9/27	34.5	(5.5–63.6)	1/27	3.3	(0.0–11.2)
Non-Hispanic White	114/312	33.7	(30.3–37.2)	193/312	65.1	(61.1–69.0)	5/312	1.2	(0.0–2.3)
Non-Hispanic Black	59/132	44.1	(35.7–52.4)	67/132	51.5	(42.8–60.1)	6/132	4.5	(1.1–7.8)
Other Race-Racial	16/28	49.6	(23.2–76.1)	10/28	45.2	(15.1–75.2)	2/28	5.2	(0.0–11.6)
Total	220/522	37.2	(34.4–40.1)	288/522	61.0	(57.7–64.4)	14/522	1.7	(0.8–2.7)
Served in a foreign country									
Yes	134/275	47.3	(41.2–53.4)	130/275	50.0	(43.5–56.6)	11/275	2.7	(0.9–4.6)
No	86/246	27.2	(18.4–35.9)	157/246	72.1	(63.1–81.1)	3/246	0.7	(0.0–1.7)
Missing	0/1	.	(.–.)	1/1	100	(.–.)	0/1	.	(.–.)
Total	220/522	37.2	(34.4–40.1)	288/522	61.0	(57.7–64.4)	14/522	1.7	(0.8–2.7)

**Table 4:** Distribution of Hepatitis A prevalence by selected socio-demographic characteristics and sex (Prevalence %), N = 522.

CHARACTERISTIC	MALE			FEMALE			TOTAL		
	UNWEIGHTED N/N	WEIGHTED PREV. %	95% CL	UNWEIGHTED N/N	WEIGHTED PREV. %	95% CL	UNWEIGHTED N/N	WEIGHTED PREV. %	95% CL
Age in years at screening									
20–39	36/46	86.4	(76.0–96.7)	3/6	53.3	(1.0–100)	39/52	82.4	(72.6–92.2)
40–59	42/114	31.4	(22.4–40.3)	8/24	28.4	(7.5–49.3)	50/138	30.8	(23.1–38.5)
>= 6060	127/326	29.3	(24.8–33.7)	4/6	58.3	(7.9–100)	131/332	30.1	(25.3–34.9)
Total	205/486	37.1	(33.6–40.6)	15/36	38.9	(17.3–60.4)	220/522	37.2	(34.4–40.1)
Race/Hispanic origin									
Mexican American	14/23	67.1	(41.3–92.8)	0/0	.	(.–.)	14/23	67.1	(41.3–92.8)
Other Hispanic	15/25	58.9	(24.9–93.0)	2/2	100	(.–.)	17/27	62.1	(30.6–93.6)
Non-Hispanic White	108/296	33.6	(29.2–37.9)	6/16	36.0	(9.1–62.8)	114/312	33.7	(30.3–37.2)
Non-Hispanic Black	53/115	45.8	(35.6–55.9)	6/17	37.2	(14.3–60.2)	59/132	44.1	(35.7–52.4)
Other Race	15/27	48.3	(21.5–75.2)	1/1	100	(.–.)	16/28	49.6	(23.2–76.1)
Total	205/486	37.1	(33.6–40.6)	15/36	38.9	(17.3–60.4)	220/522	37.2	(34.4–40.1)
Served in a foreign country									
Yes	127/261	46.6	(39.8–53.3)	7/14	58.2	(29.1–87.3)	134/275	47.3	(41.2–53.4)
No	78/224	27.1	(17.9–36.3)	8/22	27.6	(2.1–53.1)	86/246	27.2	(18.4–35.9)
Missing	0/1	.	(.–.)	0/0	.	(.–.)	0/1	.	(.–.)
Total	205/486	37.1	(33.6–40.6)	15/36	38.9	(17.3–60.4)	220/522	37.2	(34.4–40.1)
